# A comparison of screening tests for detection of high-grade cervical abnormalities in women living with HIV from Cameroon

**DOI:** 10.1186/s13027-020-00311-w

**Published:** 2020-07-11

**Authors:** Philip E. Castle, Rogers Ajeh, Anastase Dzudie, Ernestine Kendowo, Norbert Fuhngwa, Andre Gaetan Simo-Wambo, Denis Nsame, Enow Orock, Tiffany M. Hebert, Amanda J. Pierz, Daniel Murokora, Kathryn Anastos, Adebola Adedimeji

**Affiliations:** 1 Department of Epidemiology and Population Health, Albert Einstein College of Medicine, Bronx, NY USA; 2 Clinical Research Education, Networking and Consultancy, Yaoundé, Cameroon; 3 Limbe Regional Hospital, Limbe, Southwest Region Cameroon; 4 Department of Biomedical Sciences, University of Buea, Buea, Cameroon; 5 Brick By Brick, Kampala, Uganda

## Abstract

**Background:**

Women living with human immunodeficiency virus (WLWH), especially those living in low- and middle-income countries (LMIC), are at increased risk of cervical cancer. The optimal cervical-cancer screening strategy for WLWH has not been determined. We therefore conducted a pilot study of screening methods in WLWH living in Limbe, Cameroon.

**Methods:**

Five-hundred sixty-six WLWH, aged 25–59 years, were enrolled. After self-collecting a cervicovaginal specimen, they underwent a pelvic exam, during which a provider also collected a cervical specimen and visual inspection after acetic acid (VIA) was performed. Both self- and provider-collected specimens were tested for high-risk HPV by the Xpert HPV Test (Cepheid, Sunnyvale, CA, USA), with the residual of the latter used for liquid-based cytology. Women testing HPV positive on either specimen and/or VIA positive were referred to colposcopy and biopsies. However, because of poor attendence for follow-up colposcopy for the screen positives due to civil strife and technical issues with biopsies, high-grade cytology and/or clinical diagnosis of cancer was used as the primary high-grade cervical abnormality endpoint. Clinical performances for high-grade cervical abnormality of HPV testing and VIA for screening WLWH, and the most carcinogenic HPV genotypes and/or VIA to triage high-risk HPV-positive WLWH, were evaluated.

**Results:**

Four-hundred eighty-seven (86.0%) WLWH had results for HPV testing on both specimen, VIA, and cytology and were included in the analysis. Forty-nine (10.1%) had a high-grade cervical abnormality. HPV testing on provider- and self-collected specimens was more sensitive than VIA (95.9 and 91.8% vs. 43.8%, respectively, *p* < 0.01 for both comparisons) for identifying women with high-grade cervical abnormalities. HPV testing on provider- and self-collected specimens was less specific than VIA (57.5 and 51.6% vs. 89.7%, respectively, *p* < 0.01 for both comparisons) for identifying women with high-grade cervical abnormalities; HPV testing on provider-collected specimens was more specific than on self-collected specimens (*p* < 0.01). Among HPV-positive women, HPV16/18/45 detection or VIA positivity had a sensitivity and positive predictive value of 73.5 and 29.0%, respectively, for provider-collected specimens and 68.8 and 22.9%, respectively, for self-collected specimens for high-grade cervical abnormalities.

**Conclusions:**

HPV testing was more sensitive but less specific than VIA for detection of high-grade cervical abnormality in WLWH. Improved triage methods for HPV-positive WLWH are needed.

**Trial registration:**

NCT04401670 (clinicaltrials.gov); retrospectively registered on May 26, 2020

## Introduction

Women living with human immunodeficiency virus (HIV) (WLWH) are at a significantly higher risk of cervical cancer than women who are not infected by HIV [1–4]. Prophylactic vaccination against human papillomavirus (HPV), the obligate viral cause of virtually all cervical cancer [5], may be the ultimate cervical-cancer prevention strategy. However, there are several generations of mid-adult women, notably WLWH, who already are HPV infected and will not benefit greatly from, or even be targeted for, HPV vaccination. Thus, cervical-cancer screening will be necessary for the foreseeable future, especially for high-risk women such as WLWH.

Cameroon is a high cervical-cancer burden country in Central sub-Saharan Africa (SSA). Cameroon has age-standardized cervical cancer incidence rate of 31.3 per 100,000 (28th in the world) and mortality rate of 21.9 per 100,000 (26th in the world) [6, 7]. High rates of cervical cancer incidence and mortality are due in part to the high prevalence of HIV, which is estimated at 4.7% in 2018 [8]. To date, there is no HPV vaccination program in Cameroon, further emphasizing the need for cervical-cancer screening for those generations of women unprotected against cervical cancer.

Recent studies [9–12] comparing high-risk HPV testing, visual inspection after acetic acid (VIA), and/or Pap for the detection of cervical precancer/cancer in WLWH in SSA have found that 1) hrHPV detection was more sensitive but less specific than VIA and 2) cytology was equally or more sensitive but less specific than VIA and 3) cytology was equally or more sensitive but less specific (vs. the converse) than hrHPV testing. Results and conclusions have varied, raising the question of what is the optimal approach in WLWH living in SSA in terms of effectiveness. We therefore conducted a pilot study of different screening methods in WLWH population in Limbe, Cameroon.

## Methods

### Population

We enrolled a convenience, consecutive sample of 878 consenting women, aged 25–59 years, receiving health services at the Limbe Regional Hospital in Limbe, Cameroon from May 01, 2017 to April 26, 2018. Inclusion criteria were aged 25–59 years, confirmed to be either a WLWH or HIV [−] woman, never undergone cervical cancer screening, no history of invasive cervical cancer, and willing, and able to competently understand and provide written, informed paper-based consent to participate. Exclusion criteria were those who did not meet the inclusion criteria or were pregnant, had signs of abnormalities or non-menstrual bleeding suggestive of invasive cervical cancer, undergone hysterectomy, and/or, based on the judgment of the clinicians, were not sufficiently healthy to participate in a research study. Women were recruited according to HIV status. Of the 878 women enrolled in the study, 566 were WLWH attending HIV Treatment Center. Inclusion and exclusion criteria were previously described [13]. Institutional Review Board of Albert Einstein College of Medicine and the National Ethics Committee of Cameroon approved the study.

### Enrollment visit

Enrolled women were escorted to a private room and provided a device (Viba Brush, Rovers, Oss, The Netherlands) for self-collection and instructions on how to self-collect their own sample, which was then placed into ThinPrep® medium (PreservCyt; Hologic, Inc., Bedford, MA, USA). Women then underwent a pelvic exam, at which time they had a specimen collected using Cervex Combi Brush (Rovers) into PreservCyt for ThinPrep liquid-based cytology (LBC) and molecular testing. Finally, VIA was performed by a physician trained for this purpose.

### Laboratory testing

The Xpert HPV Test (Cepheid, Sunnyvale, CA, USA) on the GeneXpert platform was used to test 1-mL aliquots of the self-collected and provider-collected specimens for high-risk HPV per the manufacturer’s instructions [14]. Xpert HPV Test detects 14 HPV types are detected in 5 fluorescent channels: Channel 1 (HPV16), Channel 2 (HPV18 and 45), Channel 3 (HPV31, 33, 35, 52, and 58), Channel 4 (HPV51 and 59), and Channel 5 (HPV39, 56, 66, and 68). Residual provider-collected specimens in PreservCyt were used to make ThinPrep liquid-based cytology slides, which were read according to The Bethesda System [15] (Center For Disease Detection, San Antonio, TX, USA). Cytologic interpretations were categorized as 1) negative if negative for intraepithelial lesion or malignancy cytology, 2) low-grade cytologic abnormalities if atypical squamous cells of undetermined significance or low-grade intraepithelial lesion cytology, or 3) high-grade cytologic abnormalities if high-grade squamous intraepithelial lesion (HSIL), atypical squamous cells cannot rule out HSIL (ASC-H), or atypical glandular cells (AGC).

### Clinical management

Women who tested positive for HPV on either specimen or were VIA positive were referred to colposcopy that included a 4-quadrant microbiopsy protocol that included endocervical curettage (ECC) for diagnosis [16, 17]. Screen-positive women were then treated by thermal ablation or loop electrosurgical excision procedure according to WHO guidelines [18, 19].

### Analysis

The original study design proposed to use histologic endpoints for analyses. However, of the 316 WLWH who screened positive for HPV on either specimen or by VIA, only 91 had histologic results due to failure to return for colposcopy (*n* = 201), in part due to civil unrest at the time of the study, or inadequate quality of histology (*n* = 24). We therefore defined our endpoint of high-grade cervical abnormality based cytologic interpretation of high-grade cytology, as was previously done in Senegal [20, 21], and/or a clinical diagnosis of cervical cancer.

Fisher’s exact test for categorical variables and Kruskal-Wallis test for continuous variables were used to test for differences between the sub-groups included in and excluded from these analyses. We calculated the percent positive of each test for normal, low-grade, and high-grade cervical cytology. Ordinal logistic regression was used to assess whether of HIV status and median age influenced the trend of a positive screening test with increasing severity of cervical abnormalities.

Sensitivity, specificity, negative (NPV) and positive (PPV) predictive values, and odds ratio (OR) with 95% confidence intervals (95%CI) of each screening test for high-grade cervical abnormalities were calculated. Differences in sensitivity and specificity were tested for statistical significance (*p* < 0.05) using an exact version of the McNemar chi-square test.

Finally, we calculated the sensitivity and PPV with 95%CI of different triage strategies for identifying high-grade cervical abnormalities in women who tested HPV positive on the provider-collected or the self-collected specimen.

## Results

Five hundred forty-seven of the 566 (96.6%) recruited WLWH had results for all three screening tests; 6 were missing HPV testing results for the provider-collected specimens, 6 were missing HPV testing results for the self-collected specimens, and 7 VIA results were missing. Of the 547 WLWH with all three screening results, 487 (89.0% with all three screening results; 86.0% of 566 WLWH enrolled; 288 (91.1%) of 316 screen positives) also had cytology results and were included in the analysis. Those included in the analysis (with cytology results) were more (marginally) likely to screen positive overall (*p* = 0.05), tested positive for HPV on the provider-collected specimen (*p* = 0.13) and self-collected specimen (*p* = 0.01), but not VIA (*p* = 0.84), compared to those excluded from the analysis (missing cytology results). There was no difference in age (*p* = 0.27) or CD4 counts (*p* = 0.71) between those with and without cytology results.

WLWH included in this analysis had a mean, median, and age range of 42, 42, and 25–59 years, respectively, and a mean, median, and CD4 count range of 569, 544, and 13–2406 per mm^3^, respectively. Of those 487 included in this analysis, 467 (95.9%) were currently on anti-retroviral therapy at the time of enrollment. More demographic data are shown in Table 1.

**Table 1:** Sociodemographics of 487 Cameroonian women living with human immunodeficiency virus participating in this study

	N	%
**Age Group (Years)**		
< 30	23	4.7
30–39	151	31.0
40–49	223	45.8
50–59	90	18.5
**Anti-Retroviral Treatment**		
Yes	467	95.9
No	20	4.1
**Baseline CD Count (per mm**^**3**^**)**		
< 200	60	12.3
200–349	74	15.2
349–499	84	17.3
≥ 500	269	55.2
**Marital Status**		
Married/Cohabitating	178	36.6
Divorced/Separate	39	8.0
Widowed	84	17.3
Single	186	38.3
**Occupation**		
Unemployed	63	12.9
Government employed	23	4.7
Self-employed	199	40.9
Farming	79	16.2
Other/Missing	123	25.3
**Age at First Sex (Years)**		
< 16	122	25.05
16 or 17	122	25.05
18 or 19	118	24.23
≥ 20	102	20.94
Missing	23	4.72
**Number of Sexual Partners, Lifetime**		
1	27	5.5
2–4	211	43.3
5–6	105	21.6
7 or more	120	24.6
Missing	24	4.9

Forty-nine women (*n* = 49; 10.1% of 487 enrolled women) had evidence of high-grade cervical abnormalities: 47 had high-grade cytology, 1 had clinical diagnosis of cancer and high-grade cytology, and 1 had a clinical diagnosis of cancer. All three screening results were more likely to be positive with increasing severity of the cervical abnormality (*p* < 0.01 for all) (Table 2). This trend of increasing screening test positivity with increasing severity of the cervical abnormality remained significant after adjusting for CD4 and age (*p* < 0.01 for all). CD4 counts were independently and inversely associated with increasing severity of the cytologic interpretation after controlling for screen test positivity (*p* = 0.03 for both HPV test results and *p* < 0.01 for VIA). All three screening tests were positive for the two women with a clinical diagnosis of cancer.

**Table 2:** Distribution of results for provider-collected (Provider) or self-collected (Self) specimen tested for HPV or visual inspection after acetic acid (VIA) by severity of the cytologic interpretation for women living with HIV. Abbreviations: N+, number of positives; %+, percent positive

		HPV Testing: Provider					HPV Testing: Self					VIA				
**Severity of cytologic interpretation**	**N**	**N+**	**%+**	**p**	**p**_+_*	p (CD4)*	**N+**	**%+**	**p**	**p**_+_*	p (CD4)*	**N+**	**%+**	**p**	**p**_+_*	p (CD4)*
Negative	303	87	28.7	*< 0.01*	*< 0.01*	0.03	114	37.6	*< 0.01*	*< 0.01*	*0.03*	14	4.6	*< 0.01*	*< 0.01*	*< 0.01*
Low-Grade	135	99	73.3				98	72.6				31	23.0			
High-Grade	49	47	95.9				45	91.8				22	44.9			

*Results on an ordinal logistic regression model for the association of the severity of the cytologic interpretation with screening test positivity adjusted for CD4 and age; p_+_ is the *p* value for likelihood of testing positive, and p (CD4) is the p value for the likelihood of lower CD4 counts with increasing severity of the cytologic interpretation. Age was not associated with severity of cytologic interpretation

Self-collected specimens were more likely to test HPV positive than provider-collected specimens overall (*p* < 0.01). This was due to the greater likelihood of self-collected specimens testing HPV positive than provider-collected specimens among those with negative cytology (38.7% vs. 27.6%, respectively, *p* < 0.01) whereas there was no significant difference in the two specimens testing HPV positive among those who had low-grade (72.6% vs. 73.3%, respectively, *p* = 1.00) or high-grade (91.8% vs. 95.9%, respectively, *p* = 0.50) cervical abnormalities.

Among WLWH with negative cytology, the ct (cycle threshold) values of HPV-positive self-collected specimens for those whose provider-collected specimen also tested positive was lower (higher signal strength) (Self+/Provider+) than for those provider-collected specimen tested negative (Self+/Provider-) for all Xpert Channels (Supplemental Table): HPV16 (*p* = 0.01), HPV18 and 45 (*p* = 0.01), HPV31, 33, 35, 52, and 58 (*p* < 0.01), HPV51 and 59 (*p* = 0.06), and HPV39, 56, 66, and 68 (*p* < 0.01).

Table 3 shows the performance of each screening test for high-grade cervical abnormalities. There was no significant difference in the sensitivity of HPV testing of provider- vs. self-collected specimens (95.9% vs. 91.8%, respectively, *p* = 0.50) but both were more sensitive than VIA (44.9%, *p* < 0.01 for both comparisons). HPV testing of the provider-collected specimen was more specific than of the self-collected specimen (57.5% vs. 51.6%, respectively, *p* < 0.01), and both were less specific than VIA (89.7%, *p* < 0.01 for both comparisons).

**Table 3:** Sensitivity, specificity, positive (PPV) and negative (NPV) predictive values, and odds ratio (OR) with 95% confidence interval (95%CI) of the provider-collected (Provider) or self-collected (Self) specimen tested for HPV or visual inspection after acetic acid (VIA) for high-grade cervical abnormalities for women living with HIV

	Provider		Self		VIA	
	Estimate	95%CI	Estimate	95%CI	Estimate	95%CI
Sensitivity	95.9%	86.0–99.5%	91.7%	80.4–97.7%	44.9%	30.7–59.8%
Specificity	57.5%	52.8–62.2%	51.6%	46.8–56.4%	89.7%	86.5–92.4%
PPV	20.2%	15.2–25.4%	17.5%	13.1–22.7%	32.8%	21.8–44.5%
NPV	99.2%	97.2–99.9%	98.3%	95.6–99.5%	93.6%	90.8–95.7%
OR	31.8	7.6–132.7	12.0	4.2–33.9	7.1	3.7–13.5

The specificity of HPV testing for provider-collected (*p* = 0.04) and self-collected (*p* = 0.01) specimens for women with high-grade cervical abnormalities was greater for WLWH with CD4 counts of ≥350 cells/ mm^3^ compared to those with CD4 counts of < 350 cells/mm^3^. There was no appreciable difference in sensitivity stratified on CD4 counts (data not shown). Similar results were observed if the endpoint was restricting to HSIL cytology (i.e., excluding ASC-H and AGC from the endpoint) (data not shown).

Finally, we compared different triage strategies for HPV-positive result from either the provider- or self-collected specimen (Table 4). Similar clinical performance was observed for either specimen type. HPV16 with or without HPV18/45 detection AND VIA positivity resulted relatively low sensitivities (range: 20–31%) and high PPVs (range: 54–64%). For example, HPV16/18/45 detection or VIA positivity had a sensitivity and positive predictive value of 73.5 and 29.0%, respectively, for provider-collected specimens and 68.8 and 22.9%, respectively, for self-collected specimens for high-grade cervical abnormalities. By comparison, HPV16 with or without HPV18/45 detection OR VIA positivity resulted in higher sensitivities (range: 57–74%) and lower PPV (range: 24–33%). For example, HPV16/18/45 detection or VIA positivity had a sensitivity and positive predictive value of 73.5 and 29.0%, respectively, for provider-collected specimens and 69.4 and 23.6%, respectively, for self-collected specimens for high-grade cervical abnormalities.

**Table 4:** Sensitivity and positive predictive value (PPV), with 95% confidence interval (95%CI), of triage strategies for high-grade cervical abnormalities among women living with HIV who tested HPV positive on the provider-collected (Provider) or self-collected (Self) specimen for women living with HIV. Sensitivity was calculated for all endpoints diagnosed, not just among HPV-positive women. N+, number of positives by that triage strategy; N_HG_, number of high-grade cervical abnormalities interpretation who tested positive by that triage strategy

	Provider						Self					
			Se		PPV				Se		PPV	
	N+	N_**HG**_	%	95%CI	%	95%CI	N+	N_**HG**_	%	95%CI	%	95%CI
HPV+	223	47	95.9%	86.0–99.5%	20.7%	15.2–25.9%	257	45	91.8%	80.4–97.7%	17.5%	13.1–22.7%
HPV16+	47	18	36.7%	23.4–51.7%	38.3%	24.5–53.6%	57	17	34.7%	21.7–49.6%	29.8%	18.4–43.4%
HPV16/18/45+	95	30	61.2%	46.2–74.8%	31.6%	22.4–41.9%	118	29	59.2%	44.2–73.0%	24.6%	17.1–33.4%
VIA+	56	21	42.9%	28.8–57.8%	37.5%	24.9–51.5%	54	20	38.8%	25.2–53.8%	37.0%	24.3–51.3%
HPV16+ and VIA+	16	10	20.4%	10.2–34.3%	62.5%	35.4–84.8%	14	9	18.4%	8.8–32.0%	64.3%	35.4–87.2%
HPV16/18/45+ and VIA+	27	15	30.6%	18.3–45.4%	55.6%	35.3–74.5%	28	15	30.6%	18.3–45.4%	53.6%	33.9–72.5%
HPV16+ or VIA+	87	29	59.2%	44.2–73.0%	33.3%	23.6–44.3%	97	28	57.1%	42.2–71.2%	28.9%	20.1–39.0%
HPV16/18/45+ or VIA+	124	36	73.5%	58.9–85.1%	29.0%	21.2–37.9%	144	34	69.4%	54.6–81.7%	23.6%	16.9–31.4%

## Discussion

We found that HPV testing of either a provider- or self-collected specimen to be very sensitive but poorly specific for the identification of WLWH with cytologic evidence of high-grade cervical abnormalities. By comparison, VIA was much less sensitive but much more specific as called by providers in this study.

Notably, HPV testing of self-collected specimens in this study was more likely to test HPV positive as previously noted [13], and therefore was less specific for high-grade cervical abnormalities, than HPV testing of provider-collected specimens. This result contrasts with a recent meta-analysis reporting equivalent specificity for self- and provider-collected specimens [22]. The difference was not related to HIV status as we observed a similar difference in HPV positivity between sample types in WLWH and HIV-uninfected women included in this study [13].

The greater HPV positivity and therefore lower specificity of HPV testing of self-collected specimens compared to the provider-collected specimens was due to a higher HPV test positivity among lower-risk WLWH with negative cytology. Signal strengths of the HPV-positive self-collected specimens among the discordant results (Self+/Provider-) were generally lower than that of concordant HPV-positive results (Self+/Provider+). We suggest that there might have been small, focal, vaginal HPV infections that were sampled only by self-collection and tested HPV positive by Xpert. Using lower ct positive cutpoints might eliminate some of these extraneous HPV-positive results [23].

We acknowledge that a major limitation of the study was the absence of histologically confirmed endpoints. The well-known challenges of pathology diagnosis still remain in many countries [24, 25], and impacted this study, despite recent publicized efforts to address this gap [26]. However, one advantage of using cytology to define the risk of cervical cancer is that we had some disease ascertainment, albeit an insensitive one, in the entire study population rather than in a subset of women with a positive-screening result. Consequently, we may have avoided some verification biases, especially in the context of limited capacity and experience in conducting colposcopy. Still, results of this study should be considered in this context and in relative terms rather than absolute clinical performance.

Another limitation was the study population consisted of only women who sought care at the Limbe clinic and therefore may not be representative of the general population. This convenience sample excludes WLWH who do not present at clinics for a variety of reason. Therefore, this study population may have different, and quite possibly lower, carriage of HPV infection than those not included in the study, resulting in better specificity.

Finally, low-cost strategies for the management of HPV-positive WLWH remains an important research goal. VIA combined with detection of the most carcinogenic and predictive HPV genotypes offers modest performance and the decision on how to use them may depend on resources available for follow-up care and cultural sensitivities related to overtreatment vs. cancer prevention. Promising new technologies such as automated visual evaluation [27] and in vivo microscopy [28] may be better at distinguishing between clinically important and benign HPV infections, an important consideration for management of WLWH who typically have a very high prevalence of HPV [23, 29–34] that is related to the degree of HIV control [29, 32, 33, 35].

## Supplementary information

**Additional file 1: Table S1.** A comparison of ct (cycle threshold) values of HPV-positive self-collected specimens for those whose provider-collected specimen also tested positive was lower (higher signal strength) (Self+/Provider+) to those whose provider-collected specimen tested negative (Self+/Provider-) for each Xpert human papillomavirus (HPV) test channel (group) among women living with HIV who had negative cytology. Abbreviation: IQR, interquartile range, *Kruskal-Wallis.

## Data Availability

The datasets during and/or analysed during the current study available from the corresponding author on reasonable request.

## References

[1] Michaud JM, Zhang T, Shireman TI, Lee Y, Wilson IB (2020). Hazard of cervical, oropharyngeal, and anal cancers in HIV-infected and HIV-uninfected Medicaid beneficiaries. Cancer Epidemiol Biomark Prev.

[2] Grulich AE, van Leeuwen MT, Falster MO, Vajdic CM (2007). Incidence of cancers in people with HIV/AIDS compared with immunosuppressed transplant recipients: a meta-analysis. Lancet.

[3] Silverberg MJ, Lau B, Achenbach CJ, Jing Y, Althoff KN, D'Souza G (2015). Cumulative incidence of cancer among persons with HIV in North America: a cohort study. Ann Intern Med.

[4] Abraham AG, D'Souza G, Jing Y, Gange SJ, Sterling TR, Silverberg MJ (2013). Invasive cervical cancer risk among HIV-infected women: a north American multicohort collaboration prospective study. J Acquir Immune Defic Syndr.

[5] Schiffman M, Castle PE, Jeronimo J, Rodriguez AC, Wacholder S (2007). Human papillomavirus and cervical cancer. Lancet.

[6] Ferlay J, Colombet M, Soerjomataram I, Mathers C, Parkin DM, Pineros M (2019). Estimating the global cancer incidence and mortality in 2018: GLOBOCAN sources and methods. Int J Cancer.

[7] IARC Global Cancer Observatory (GCO) (2018). GLOBOCAN 2018 database.

[8] United Nations AIDS Program (2020). Cameroon HIV statistics.

[9] Chung MH, McKenzie KP, De VH RBA, Rana F, Pamnani R (2013). Comparing pap smear, via, and hpv cervical cancer screening methods among hiv-positive women by immune status and antiretroviral therapy. AIDS.

[10] Firnhaber C, Mayisela N, Mao L, Williams S, Swarts A, Faesen M (2013). Validation of cervical cancer screening methods in HIV positive women from Johannesburg South Africa. PLoS One.

[11] Dartell MA, Rasch V, Iftner T, Kahesa C, Mwaiselage JD, Junge J (2014). Performance of visual inspection with acetic acid and human papillomavirus testing for detection of high-grade cervical lesions in HIV positive and HIV negative Tanzanian women. Int J Cancer.

[12] Mabeya H, Khozaim K, Liu T, Orango O, Chumba D, Pisharodi L (2012). Comparison of conventional cervical cytology versus visual inspection with acetic acid among human immunodeficiency virus-infected women in Western Kenya. J Low Genit Tract Dis.

[13] Adedimeji A, Ajeh R, Dzudie A, Kendowo E, Fuhngwa N, Nsame D (2020). Cervical human papillomavirus DNA detection in women living with HIV and HIV-uninfected women living in Limbe, Cameroon. J Clin Virol.

[14] World Health Organization (2018). Product: Xpert® HPV (WHO reference number: PQDx 0268-070-00).

[15] Solomon D, Davey D, Kurman R, Moriarty A, O'Connor D, Prey M (2002). The 2001 Bethesda system: terminology for reporting results of cervical cytology. JAMA.

[16] Pretorius RG, Zhang WH, Belinson JL, Huang MN, Wu LY, Zhang X (2004). Colposcopically directed biopsy, random cervical biopsy, and endocervical curettage in the diagnosis of cervical intraepithelial neoplasia II or worse. Am J Obstet Gynecol.

[17] Preventive Oncology International four-quadrant microbiopsy protocol. Ref Type: Internet Communication. 2014. http://www.poiinc.org/resources/poi-microbiopsy-protocol-and-instrument/.

[18] Santesso N, Mustafa RA, Schunemann HJ, Arbyn M, Blumenthal PD, Cain J (2016). World Health Organization guidelines for treatment of cervical intraepithelial neoplasia 2-3 and screen-and-treat strategies to prevent cervical cancer. Int J Gynaecol Obstet.

[19] World Health Organization (2019). WHO guidelines for the use of thermal ablation for cervical pre-cancer lesions.

[20] Hawes SE, Critchlow CW, Faye Niang MA, Diouf MB, Diop A, Toure P (2003). Increased risk of high-grade cervical squamous intraepithelial lesions and invasive cervical cancer among African women with human immunodeficiency virus type 1 and 2 infections. J Infect Dis.

[21] Hawes SE, Critchlow CW, Sow PS, Toure P, N'Doye I, Diop A (2006). Incident high-grade squamous intraepithelial lesions in Senegalese women with and without human immunodeficiency virus type 1 (HIV-1) and HIV-2. J Natl Cancer Inst.

[22] Arbyn M, Smith SB, Temin S, Sultana F, Castle P (2018). Detecting cervical precancer and reaching underscreened women by using HPV testing on self samples: updated meta-analyses. BMJ.

[23] Kuhn L, Saidu R, Boa R, Tergas A, Moodley J, Persing D (2020). Clinical evaluation of modifications to a human papillomavirus assay to optimise its utility for cervical cancer screening in low-resource settings: a diagnostic accuracy study. Lancet Glob Health.

[24] Adesina A, Chumba D, Nelson AM, Orem J, Roberts DJ, Wabinga H (2013). Improvement of pathology in sub-Saharan Africa. Lancet Oncol.

[25] Roberts DJ, Wilson ML, Nelson AM, Adesina AM, Fleming KA, Milner D (2012). The good news about cancer in developing countries--pathology answers the call. Lancet.

[26] Milner DA, Holladay EB (2018). Laboratories as the Core for health systems building. Clin Lab Med.

[27] Hu L, Bell D, Antani S, Xue Z, Yu K, Horning MP (2019). An observational study of deep learning and automated evaluation of cervical images for cancer screening. J Natl Cancer Inst.

[28] Hunt B, Fregnani JHT, Schwarz RA, Pantano N, Tesoni S, Possati-Resende JC (2018). Diagnosing cervical neoplasia in rural Brazil using a mobile van equipped with in vivo microscopy: a cluster-randomized community trial. Cancer Prev Res (Phila).

[29] Menon S, Rossi R, Zdraveska N, Kariisa M, Acharya SD, Vanden Broeck D (2017). Associations between highly active antiretroviral therapy and the presence of HPV, premalignant and malignant cervical lesions in sub-Saharan Africa, a systematic review: current evidence and directions for future research. BMJ Open.

[30] Taku O, Businge CB, Mdaka ML, Phohlo K, Basera W, Garcia-Jardon M (2020). Human papillomavirus prevalence and risk factors among HIV-negative and HIV-positive women residing in rural eastern cape, South Africa. Int J Infect Dis.

[31] Castle PE, Varallo JE, Bertram MM, Ratshaa B, Kitheka M, Rammipi K (2020). High-risk human papillomavirus prevalence in self-collected cervicovaginal specimens from human immunodeficiency virus (HIV)-negative women and women living with HIV living in Botswana. PLoS One.

[32] Murenzi G, Kanyabwisha F, Murangwa A, Kubwimana G, Mutesa L, Burk RD (2020). Twelve-year trend in the prevalence of high-risk human papillomavirus infection among Rwandan women living with HIV. J Infect Dis.

[33] Kelly HA, Sawadogo B, Chikandiwa A, Segondy M, Gilham C, Lompo O (2017). Epidemiology of high-risk human papillomavirus and cervical lesions in African women living with HIV/AIDS: effect of anti-retroviral therapy. AIDS.

[34] Clifford GM, Tully S, Franceschi S (2017). Carcinogenicity of human papillomavirus (HPV) types in HIV-positive women: a meta-analysis from HPV infection to cervical cancer. Clin Infect Dis.

[35] Kelly H, Weiss HA, Benavente Y, de Sanjose S, Mayaud P (2018). Association of antiretroviral therapy with high-risk human papillomavirus, cervical intraepithelial neoplasia, and invasive cervical cancer in women living with HIV: a systematic review and meta-analysis. Lancet HIV.

