# Supplementary material for: A comparison of screening tests for detection of high-grade cervical abnormalities in women living with HIV from Cameroon
Source: Infect Agent Cancer. 2020 Jul 11;15:45. doi: 10.1186/s13027-020-00311-w (PMC7353796; doi:10.1186/s13027-020-00311-w)
Supplement: Supplementary file 1 — Additional file 1: Table S1. A comparison of ct (cycle threshold) values of HPV-positive self-collected specimens for those whose provider-collected specimen also tested positive was lower (higher signal strength) (Self+/Provider+) to those whose provider-collected specimen tested negative (Self+/Provider-) for each Xpert human papillomavirus (HPV) test channel (group) among women living with HIV who had negative cytology. Abbreviation: IQR, interquartile range, *Kruskal-Wallis. [file 13027_2020_311_MOESM1_ESM.docx]

**Supplemental Table 1**. A comparison of ct (cycle threshold) values of HPV-positive self-collected specimens for those whose provider-collected specimen also tested positive was lower (higher signal strength) (Self+/Provider+) to those whose provider-collected specimen tested negative (Self+/Provider-) for each Xpert human papillomavirus (HPV) test channel (group) among women living with HIV who had negative cytology. Abbreviation: IQR, interquartile range

|  | Self+/Provider+ | | Self+/Provider- | |  |
| --- | --- | --- | --- | --- | --- |
| HPV Group | N+ | ct values  Mean; Median; IQR | N+ | ct values  Mean; Median; IQR | p* |
| HPV16 | 14 | 30.6; 30.9; 27.0-33.4 | 6 | 35.0; 37.2; 32.9-36.5 | 0.01 |
| HPV31/33/35/52/58 | 19 | 31.0; 30.9; 27,5-35.3 | 9 | 37.3; 36.9; 34.2-38.9 | 0.01 |
| HPV18/45 | 39 | 32.8; 32.0; 28.2-35.3 | 13 | 36.5; 36.9; 36.3-38.9 | <0.01 |
| HPV51/59 | 20 | 30.0; 30.6; 27.5-32.1 | 2 | 35.9; 35.9; 34.6-37.2 | 0.06 |
| HPV39/56/66/68 | 29 | 31.4; 30.2; 28.1-36.3 | 10 | 37.0; 37.2; 36.6-37.4 | <0.01 |

*Kruskal-Wallis
